# Sudan Dye Adulteration in Foods: A Comprehensive Review of Health Risks, Detection Methods, Regulatory Responses, and Preventive Strategies

**DOI:** 10.3390/foods15203619

**Published:** 2026-10-11

**Authors:** Te-Wen Chiu, Wen-Chang Chang, Chung-Cheng Li, Chih-Yao Hou, Ping-Hsiu Huang

**Affiliations:** 1 Department of Food Sciences, College of Life Science, National Chiayi University, No.300 Syuefu Rd., Chiayi 600355, Taiwan; taiwansealing@gmail.com (T.-W.C.); wcchang@mail.ncyu.edu.tw (W.-C.C.); 2 Mr. PEPPER Co., Ltd. No.39.ALY 1, Ln 230 Jhong Jheng Rd Dalin township, Chiayi 622002, Taiwan; 3 Food and Drug Administration, Ministry of Health and Welfare, 2F, No.180, Ziyou 2nd Rd., Zuoying Dist., Kaohsiung 813021, Taiwan; chungcheng@fda.gov.tw; 4 Department of Seafood Science, College of Hydrosphere, National Kaohsiung University of Science and Technology, Kaohsiung 81157, Taiwan

**Keywords:** cross-contamination, health, food fraud, food supply, sustainability

## Abstract

Sudan dyes (SDs), synthetic azo dyes used exclusively in industrial applications, are frequently detected as illegal adulterants in spice products due to their vivid color and low cost. This review systematically assesses the health risks posed by SD exposure and characterizes global contamination patterns across complex spice supply chains. These supply chains are inherently complex, presenting risks such as contamination, adulteration, and food fraud (FF). Mitigating these risks demands a coordinated approach: integrating advanced technology, enforcing regulatory compliance, and embedding sustainability across the entire chain. Key actions include deploying scalable detection tools, delivering targeted training, and aligning with international standards to strengthen transparency and traceability. Regulatory challenges stem from supply chain complexity, varying international standards, and enforcement limitations in producing regions. However, adopting cutting-edge tools, enhancing supply chain visibility, and promoting sustainable practices can reduce contamination, adulteration, and fraud, strengthening food safety and resilience. In summary, this review emphasizes the need for integrated prevention strategies combining blockchain-based traceability, Internet of Things (IoT) monitoring, harmonized global regulations, and strengthened supplier oversight to safeguard food authenticity and public health.

## 1. Introduction

### 1.1. Food Coloration: Functions and Classifications

During the development or manufacturing of processed foods, maximum color protection is achieved, and safe (regulator-approved), intense, and vibrant colorants (natural or synthetic color additives) are utilized [1,2]. Color is widely used in food and cosmetics marketing to enhance product appeal by improving visual aesthetics, palatability, and clarity of information [1,3,4]. Color strongly influences purchasing decisions; it acts as a visual cue that shapes perceptions of aesthetic quality, even when unrelated to objective product attributes [1,2,3,5,6]. Food colors function via chromophoric groups, such as azo dyes (AZD), azoxy, carbonyl, nitro, and thiocarbonyl dyes, and differ from pigments in that they chemically bond to substrates [5,7]. They are classified as either synthetic (containing exo- and chromophoric groups) or natural. [5,7]. While natural colorants help maintain the original color of food products, they remain highly vulnerable to the high temperatures used in food manufacturing, which can adversely affect their sensory qualities (color and flavor) [2,8,9]. Furthermore, natural colorings derived from fruits’ distinctive flavors may not be universally suitable for all processed foods [2,8].

### 1.2. Global Production and Applications of AZD and SD

The global synthetic dye industry produces over 700,000 tons annually, half to four-fifths of which are AZD (containing the –N=N– chromophore) [10,11,12,13]. In addition, industries such as plastics, polyester, nylon, leather (wool), cosmetics, ink, paint, paper, and food manufacturing all involve substantial use of dyes [7,14,15,16]. Remarkably, the global market for synthetic dyes (including AZD) may reach USD 14.8 billion by 2032 [17]. Globally, the AZD market value from 2025 to 2026 would grow from USD 6.03 to 6.39 billion, then continue to increase at a compound annual growth rate (CAGR) of 6.37%, potentially reaching USD 9.31 billion by 2032 [18]. While this underscores the industry’s growing momentum, it also signals an escalating environmental burden from pollutant emissions associated with synthetic dyes (60–80% of which are AZD) [15,17,19,20]. Regarding the dye wastewater discharge, the ranks are as follows: textile industry (55%), dyeing industry (21%), paper and pulp industry (9%), tanneries and paint industry (8%), and dye manufacturing (7%) [21]. However, these AZDs are widely used in a variety of industries, including cosmetics, food and beverages, leather tanning, paper, plastics, printing inks, and textile dyeing [11,18,22]. Currently, roughly 65–70% of AZDs serve as food additives due to their versatility, cost-effectiveness, accessibility, stability, consistency, and lack of off-flavors (disagreeable odors), providing abundant vivid colors [8,9,23]. Consequently, AZDs are widely used in beverages, candies, ice cream, ketchup, jams, jellies, sausages, baked goods, chewing gum, canned foods, and pickles [5,8,23,24,25,26]. The following AZDs are approved as food additives, each with an Acceptable Daily Intake (ADI) of 0.15–7.5 mg/kg body weight (BW): Allura Red AC (E129, 7.0), Amaranth (E123, 0.15–0.8), Brilliant Blue (E133, 6.0), Brown HT (E155, 1.5), Carmoisine (E122, 4.0), Ponceau 4R (E124, 0.7), Sunset Yellow (E110, 4.0), and Tartrazine (E102, 7.5), all values in mg/kg BW [5,8,9,24,27]. The most commonly used include tartrazine, sunset yellow, and azorubine, which exhibit excellent coloring performance related to their chemical structures [8,9]. It is undeniable that the widespread use of synthetic AZDs and their associated compounds is linked to reduced gut microbiota diversity, allergic reactions, organ toxicity, cytotoxicity, mutagenicity, carcinogenicity, genotoxicity, and attention deficit hyperactivity disorder (ADHD), raising serious public health concerns [2,3,5,9,22,24,28]. Simultaneously, human exposure to these insoluble AZD also arises from direct contact with everyday consumer products (such as paper, plastics, polymers, surface coatings, etc.) [19]. However, SDs have been universally banned from food applications owing to robust evidence of their carcinogenicity.

### 1.3. Food Safety Risks and International Regulatory Differences

Despite varying international regulations and lingering safety concerns regarding ADI and permissible additive levels, AZD maintains distinct technical and economic advantages over natural colorants in these industries [5,8,18,29]. International food colorant regulations are stringent and multifaceted, compelling manufacturers to ensure the safety of their color additives and to comply with the legal standards of every market they enter [30]. It also indicates that identical food colorings, manufactured with the same formulation and intended for the same uses, can be approved in one region but prohibited in another [8,30,31]. For example, the European Food Safety Authority (EFSA) sets an ADI for Sunset Yellow (E110), while several European countries and the European University Association have banned it in food; Finland and Norway classify it as a carcinogen [32]. Furthermore, currently permitted food coloring E110 serves as a sulfonated version of the banned SD I; namely, due to limitations in production technology, SD I may still be present as an impurity [32,33]. In another industrial application, AZD, dimethyl yellow (an oil-soluble pH indicator) has been categorized by the International Agency for Research on Cancer (IARC) as a Group 2B human carcinogen [34]. It is frequently used to adulterate foods such as butter and curry products. The Hong Kong Food and Drug Administration detected dimethyl yellow in tofu and tofu skin in December 2014, and adulterated products were recalled in Taiwan [34]. Over 40 companies were implicated in this incident, with more than 2 tons of products destroyed. The involved businesses face up to seven years’ imprisonment and fines of up to New Taiwan dollars (NTD) 80 million [35]. These events also prompted the TFDA to develop a series of analytical methods for identifying dimethyl yellow and diethyl yellow in food, including a liquid chromatography-tandem mass spectrometry (LC-MS/MS) method [34,36]. Conventional detection methods such as chromatography, spectrophotometry, voltammetry, and capillary electrophoresis were time-consuming, costly, and cumbersome, making them unsuitable for rapid detection and production-site monitoring [22,27,33,37,38,39,40]. Farmers, food manufacturers, industrialists, health professionals, and researchers are increasingly seeking affordable instruments that enable accurate, time-efficient quality assessments of food and agricultural products [41]. Nevertheless, numerous scientists have devoted their efforts to machine learning (ML) driven data visualization, with convolutional neural network (CNN) models, pioneering electrochemical and nanosensors as straightforward, rapid, and sensitive tools for combating FF [22,27,33,37,41,42,43]. Furthermore, it is essential to integrate the development of environmentally sustainable analytical approaches, including the use of eco-friendly solvents, reduced energy consumption, and minimized waste [44,45,46]. Therefore, this review aimed to highlight the health risks associated with AZD (particularly SDs, which are prohibited in food applications) and to evaluate potential causes of trace residues in food ingredients. It illuminates under-examined dimensions of their negative consequences, a domain that has thus far garnered scant scholarly and public attention.

## 2. Legal Prohibition and Health Risks of SDs in Food

### 2.1. Illegal SDs Adulteration in Food: Carcinogenicity and Health Risks

**Figure 1:**
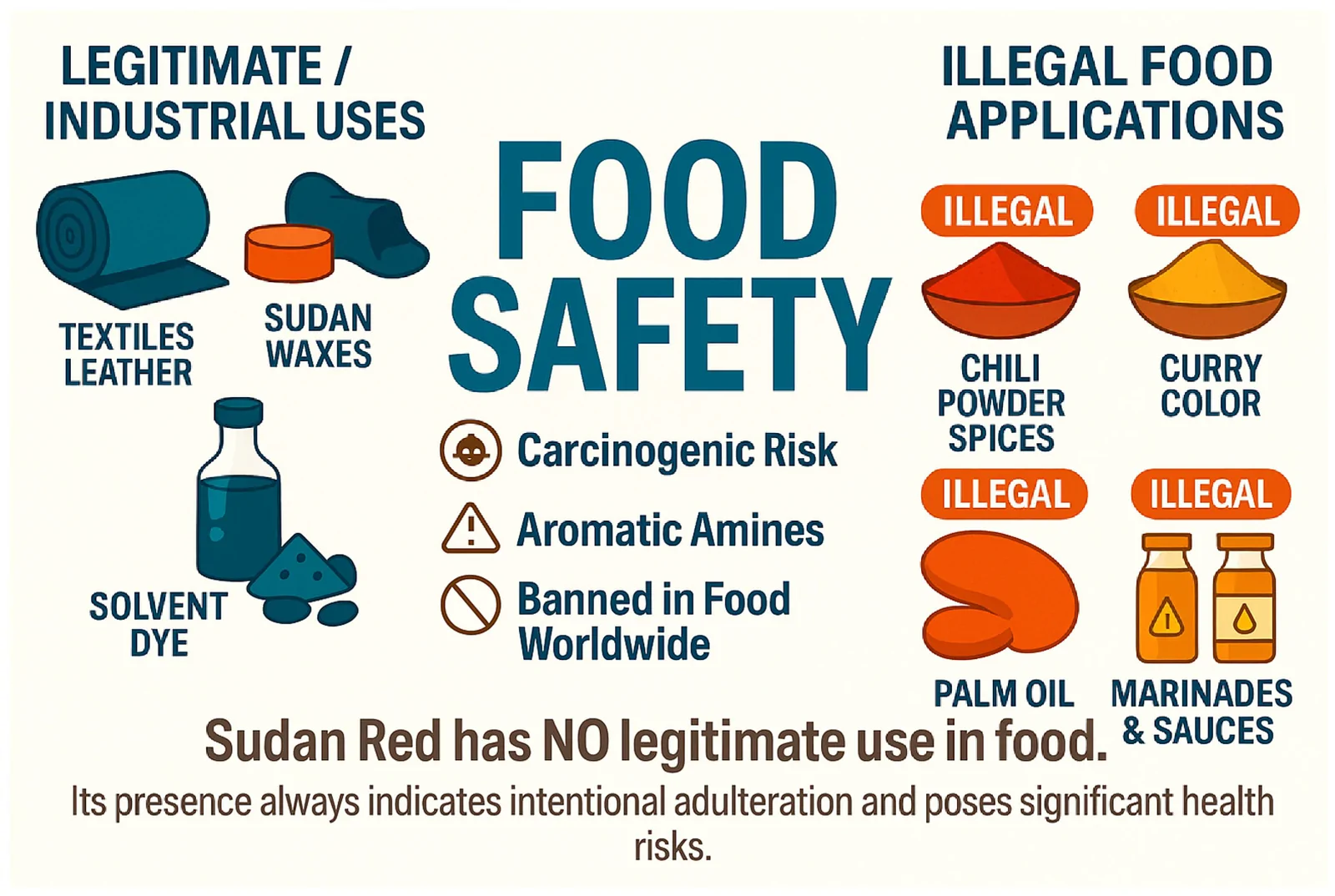
Dual nature of Sudan dyes (SDs): legitimate industrial applications versus illegal food adulteration. The dual nature of SDs, legitimate industrial utility versus illegal food application, is illustrated in Figure 1, which categorizes SD applications into two domains. On the industrial side, SDs serve as colorants for petroleum products, shoe polishes, solvents, plastics, and waxes, representing their in tended use under regulatory exemption controls. In contrast, their detection in food matrices (chili powder, curry blends, palm oil, salted duck eggs) represents illegal adulteration driven by economic motivation. This figure underscores the fundamental challenge of SD regulation: distinguishing between legitimate industrial dye flows and illicit diversion into food supply chains requires robust traceability systems and borders.

SD’s vivid red-orange color makes it a target for unscrupulous merchants who illegally add it to spices and seasonings (such as chili powder, paprika, and curry) (Figure 1) [47]. SD I–IV have been listed as Group 3 carcinogens by the IARC, while these dyes have been globally banned from use in the food supply chain to strengthen market oversight and protect public health [36,48,49,50,51]. In particular, SD has been implicated in tumor development in human organs such as the liver, bladder, and spleen [46,52]. However, SDs were widely used in industrial applications (for dyeing goods, excluding food and cosmetics) and in scientific applications (as colorants for tracers or indicators) [48,49]. It is crucial to prevent industrial dyes from entering the food supply chain, as their unauthorized use has frequently violated food safety regulations [44,48]. At the same time, illegal additives such as SDs have drawn significant attention from global regulatory authorities, with their detection typically leading to product recalls, fines, destruction, or even criminal prosecution [53]. The distinction between legitimate industrial use and illegal food application of SDs, as illustrated in Figure 1, encapsulates the fundamental regulatory challenge: SDs are legally manufactured and traded for industrial purposes but illegally introduced into food supply chains through diversion, mislabeling, or deliberate adulteration.

SDs are synthetic AZDs that dissolve in hydrocarbons, oils, fats, plastics, shoe polishes, and waxes for coloring purposes [22]. These compounds hydrolyze to amines (such as benzidine, aromatic amines, 2-naphthylamine, etc.) in the body, some of which are carcinogenic [SD I–IV, and para red (chemical structure similar to SDI)] [19,23,26,28,53]. Consequently, all countries worldwide strictly prohibit the entry of SDs into the food chain, allowing them to be used solely as industrial dyes (in biological samples, textiles, leather, plastics, and solvents), meaning only for non-food applications [13,22,49,54,55]. However, illegal use of food additives mainly occurs when unscrupulous operators deliberately add industrial-grade SD to foods such as spices, sauces, and candies [22,55,56,57,58]. Specifically, the earliest disclosure occurred in the European Union in 2003, when authorities discovered the illegal use of SD I–IV (2.8 and 3500 mg/kg chili powder) in imported chili peppers and products [22,59,60]. Cases of palm oil adulteration (employing SD IV) have been common due to consumer preference for red coloration [40]. According to the EU’s Rapid Alert System for Food and Feed (RASFF), incidents peaked in 2004–2005 (70 and 43 cases, respectively), then declined markedly from 2006 to 2022, likely due to improved public awareness and strengthened monitoring [40]. However, despite the decline in incidence, the issue of SDs adulterating palm oil persisted between 2004 and 2022, primarily originating from Africa [40]. Given these findings, all major regulatory agencies, including EFSA, FDA, Health Canada, and the TFDA, have established zero-tolerance policies for SDs in food, permitting their use exclusively in non-food industrial applications [13,22,49,54,55].

### 2.2. Global Contamination Cases: Geographic Distribution, Criminal Intent, and Pollutant Concentrations

In February 2005, the UK initiated its largest food recall to date—470 products contaminated with unauthorized SD I spanning spices (paprika, turmeric, sumac), condiments (chili oil, spicy sauces, Worcestershire sauce), and seasonings (curry powder, palm oil) [22,47,61]. The ongoing issue of illegal additions of SDs (carcinogenic AZD) as food additives remains a significant concern [62,63]. Notably, in 2006, SD IV was identified in duck and chicken eggs in mainland China [64]; in 2017, SD IV was detected in palm oil sold in markets in Ghana [53]. Mishra et al. [65] tested 800 chili powder samples from the Indian market: 532 unbranded loose products contained SD I (and trace SD IV), while all 100 branded packaged samples were free of both dyes. In contrast, all 100 branded packaged samples were free of Sudan dyes. In 2017, Taiwan reported illegal use of SD I–IV, Red G, Orange G, Black B, and Red 7B in chili products, salted duck eggs, and salted egg yolks to enhance color [66]. In 2022, Sadef et al. [67] tested 474 spice samples from Lahore, Pakistan: 241 red chili peppers and 233 turmeric samples (150 g each), and found Sudan dyes in 67% of red chili and 27% of turmeric samples.

From 2017 to 2024, Taiwan’s contamination cases were traced primarily to palm oil and chili products imported from Ghana and Turkey; authorities now conduct batch-by-batch border inspections [52]. In late October 2025, Taiwan found SD IV in several well-known cosmetic brands. A Singapore-based manufacturer, Campo Research Pte Ltd., sourced defective raw materials from mainland China and supplied them to 12 domestic cosmetic companies, impacting 20 products [68]. Similarly, Ghuniem et al. [48] tested 80 commercial spice samples in Egypt, including hot chili, paprika, curry powder, and cumin, and detected SD I–IV in all. Metal content and pesticide residues remained within safe limits. The same authors reported Sudan dye levels exceeding the limit of quantification in all chili and curry samples (range: 3.3–8709.3 mg/kg); para red (3.7–6.0 mg/kg) was detected in six samples. These findings suggest deliberate, illegal addition of Sudan dyes by unscrupulous merchants to enhance color, especially SD IV in paprika, likely due to its superior color stability and oil solubility [48]. Consequently, it serves as a better adulterant than SD I, which is more readily traceable [48]. Fortunately, no evidence to date indicates direct, immediate, or long-term harm to consumers from SD-contaminated foods. However, visual color enhancement requires only 100–1000 mg/kg, while unbranded chili powder contains 200–11,700 mg/kg [65,69]. A study in Karachi, Pakistan, found that non-branded spices contained excessive levels of SD I–IV, ranging from 1.5 to 8460 mg/kg [70]. In contrast, no SD were present in branded samples [70]. These contamination cases illustrate the supply chain complexity described in Section 2.1 and Figure 1: SDs originating from industrial dye manufacturing channels are diverted into food supply chains through intermediary traders, blenders, and exporters who may lack awareness of upstream contamination or, in cases of deliberate adulteration, actively conceal the presence of prohibited substances [47,52,61]. The concentration levels detected in various incidents, ranging from trace amounts (ppb) to significant concentrations exceeding regulatory thresholds by orders of magnitude, reflect both the intent of adulterators and the effectiveness (or lack thereof) of pre-market screening [65,67]. Surprisingly, these illegal additives, detected at levels below 100 ppm, are used not for coloring but to adulterate products and increase their weight or spiciness. Alternatively, it can be hypothesized that a grey market within the industry provides materials specifically for FF, used in chili-based products to provide spiciness, extra weight, and a red hue. However, the geographic distribution of SD contamination incidents reveals a pattern spanning developed and developing regions, reflecting both detection capacity and the globalized nature of spice supply chains [22,52,61,62,63,64,65,66,67,68]. Although SD adulteration shares economic drivers and supply chain vulnerabilities with broader FF, the following sections first cover universal detection and mitigation technologies, then focus on spice-specific prevention strategies.

## 3. Regulation and Mitigation of Illegal SD Use in Food: Science- and Policy-Based Strategies for Consumers, Producers, and Authorities

### 3.1. FF: Definition, Economic Impact, and the Associated Regulatory Challenges and Resource Limitations

The transition from subsistence agriculture to a market-driven global food trading system has firmly embedded FF within the modern food risk framework [71]. FF is the deliberate substitution, addition, tampering, or misrepresentation of food products, ingredients, or packaging, and is driven primarily by the pursuit of economic gain and the exploitation of vulnerabilities within food supply chains [34,41,72,73,74,75,76,77]. The global economic cost of FF is estimated at USD 30 billion annually, encompassing activities such as counterfeiting, adulteration, substitution, dilution, false origin labeling, document forgery, and intellectual property rights violations [43].

Case studies and examples reveal that diverse forms of FF and adulteration (including high-value-added, large-price-gaps, mass-produced, and imported/exported foods) constitute highly lucrative criminal operations [74,77,78,79,80]. While most food enterprises avoid FF due to reputational and legal risks, opportunists exploit regulatory gaps, such as inconsistencies in international import rules to profit [38,71]. Many countries lack sufficient resources to monitor spice adulteration—a widespread form of FF [81]. Where specific regulatory limits are absent, authorities typically adopt their trading partners’ maximum limits to facilitate trade [82]. The spice industry, a primary matrix for SD contamination, exemplifies the structural vulnerabilities that enable FF.

Global spice supply chains are characterized by extensive geographic dispersion (often spanning multiple continents from cultivation to final retail), numerous intermediaries (collectors, brokers, traders, agents, exporters, processors), and prolonged distribution processes [77,79,82,83]. This complexity enables adulteration in multiple ways: outdoor drying exposes spices to environmental contaminants; inadequate refrigeration during transport compromises integrity [84]; and grinding whole spices into powders masks origin, facilitating SD addition [47,74,78].

Moreover, developing countries characterized by political instability, limited regulatory capacity, and rapid population growth are disproportionately vulnerable to FF and SD adulteration [80,82,85]. The crime triangle framework, involving motivated offenders (adulterators), suitable targets (complex supply chains), and the absence of capable guardians (weak enforcement), explains why SD contamination persists despite universal prohibition [75]. Eliminating any single element of this triangle proves insufficient; effective mitigation requires simultaneous intervention across all three dimensions [75,85]. In contrast, SD adulteration exemplifies economically motivated adulteration (EMA)—a FF subtype driven solely by financial gain rather than public health intent [79,82,86]. While chronic exposure to carcinogenic SD is associated with well-documented, severe health consequences, perpetrators deliberately introduce prohibited or lower-cost substances to improve appearance, inflate weight, or reduce production costs [36,48,49,50,51,79,82,86]. In the specific context of SD contamination, adulterators exploit the visual appeal and color-stabilizing properties of SDs to mask inferior quality in spices such as chili powder, paprika, and turmeric, products that undergo extensive processing (drying, grinding, blending), making origin verification particularly challenging [47,74,78]. It is essential to distinguish FF from other categories of food safety incidents. Food safety incidents typically arise from accidental contamination introducing chemical, biological, or physical hazards [75,76]; whereas food defense involves deliberate acts intended to cause harm (e.g., terrorism) [75,80]. This distinction has important regulatory implications: FF prevention requires market-based interventions (traceability, supply chain integrity, economic incentives for compliance) rather than solely hazard-based approaches [71,75].

### 3.2. Digital Technologies and Artificial Intelligence (AI) in Food Safety Monitoring

Addressing SD adulteration requires robust monitoring and regulatory systems capable of detecting prohibited substances across complex global supply chains. Both developed and developing regions face distinct challenges in food safety control, including staffing shortages, weak enforcement capacity, digital divides, and administrative inefficiencies [87,88,89]. Hasani et al. [90] also note that FF arises from distrust among food supply chain actors, requiring authenticity verification through testing, which may expand as traceability and geographic certification systems advance [91]. The use of radio frequency identification (RFID) devices and digital traceability in the food industry remains limited, which is concerning given the prevalence of FF, yet enhancing these technologies could significantly improve supply chain transparency and integrity [79].

AI and ML are transforming food safety monitoring by enabling predictive analytics and real-time decision-making. Jin et al. [92] observed that big data applications, though still emerging, are already transforming food safety governance by enabling predictive risk assessment, optimizing sampling strategies, and supporting real-time supply chain monitoring. Mu et al. [93] highlighted the integration of AI, big data, and IoT to proactively address climate-driven food safety risks. However, implementation challenges persist in low- and middle-income countries due to limited data infrastructure and connectivity [85,93,94]. According to Kumar et al. [95], it is also necessary to incorporate quantitative assessments of energy efficiency and carbon footprint into the Sustainable Development Goals (SDGs). To effectively address climate change, advance renewable energy, and ensure equitable energy access, relevant technologies require standardized data and collaborative mechanisms [95]. Zhao et al. [96] also found that promoting blockchain-based food traceability systems (BFTS) is driven by government incentives (project returns, subsidies) and supply chain data collaboration (positive effect), rather than coordination costs (negative effect). However, addressing these issues is essential to ensure the integrity of food products and protect the health and safety of consumers. Although experimental analysis is a vital tool, it is insufficient on its own to effectively combat illegal food adulteration [75]. A comprehensive strategy must integrate robust corporate governance, stringent raw material controls, standardized manufacturing processes—aligned with the Codex Alimentarius (Codex) General Principles of Food Hygiene (GPFH) and Hazard Analysis and Critical Control Point (HACCP) frameworks—and rigorous regulatory oversight [34,72,90,97].

Moreover, HACCP’s evolution from reactive compliance to proactive, data-driven governance highlights the need for an adaptive, integrated food safety management system capable of meeting 21st-century challenges [95]. Although no single model guarantees success, effective anti-fraud initiatives require legal authority, institutional independence, technological capability, and sustained political commitment to address fraud decisively [98]. Apart from the above-mentioned monitoring and regulation, promoting education programs has proven effective in increasing consumers’ knowledge and, consequently, their awareness of food quality and authenticity [67,81,99,100,101,102]. Ultimately, the above outcome can be attributed to heightened public awareness, which motivates consumers to decline purchasing dubious (FF) products and actively mitigate health-related risks [103].

### 3.3. Supply Chain Transparency and International Trade Challenges

**Figure 2:**
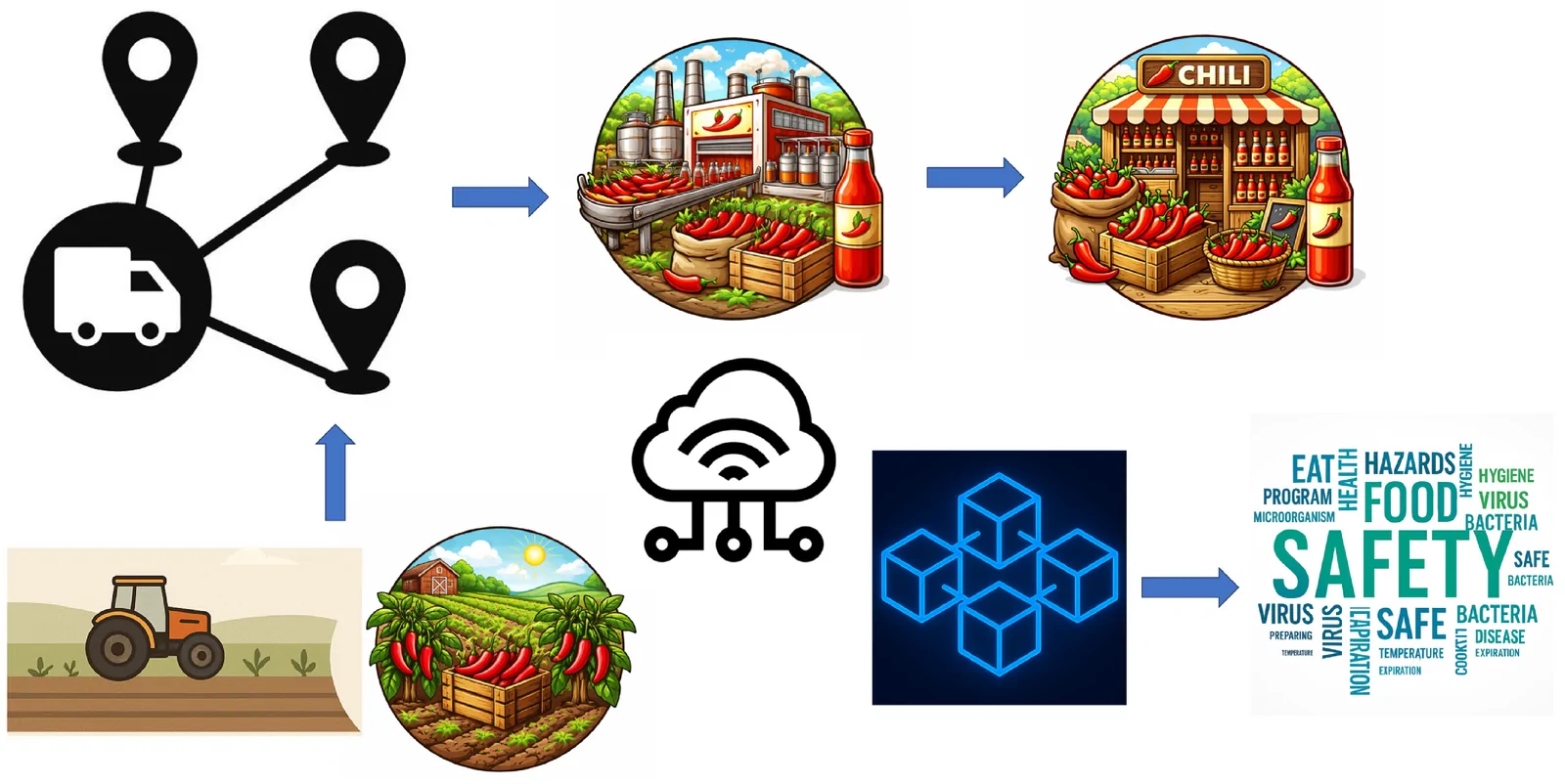
Integration of Blockchain and the Internet of Things (IoT) within a Cultivation-to-Shelf framework. Blockchain technology provides immutable, tamper-proof traceability records across all stages of the supply chain, while IoT sensors enable real-time monitoring of environmental parameters (temperature, humidity, storage conditions) during transit and production.

FF, including SD adulteration, poses distinct challenges relative to traditional food safety risks: potential adulterants are highly diverse and often unforeseen, while existing regulatory systems lack comprehensive databases of unknown contaminants, thereby requiring novel identification approaches [73,74,75,80]. Digitizing supply chain information from farm to table strengthens transparency, enables end-to-end traceability, and improves operational efficiency throughout food production and distribution [104]. Integrating big data analytics, blockchain, and IoT enables early detection of food safety threats, ensures tamper-evident traceability, and supports real-time monitoring across the supply chain (Figure 2) [89,105,106,107,108,109,110].

Notably, traditional laboratory-based analytical methods, such as LC-MS/MS, HPLC, and ELISA, remain the gold standard for SD detection but are limited by cost, turnaround time, and requirement for specialized personnel and infrastructure [52,61,106]. From an analytical perspective, no single detection method satisfies all requirements for routine monitoring: HPLC-MS/MS remains the gold standard for confirmatory analysis but is impractical for high-throughput screening; rapid methods (SERS, electrochemical biosensors, lateral flow immunoassays) offer field-deployable alternatives but face challenges in reproducibility, standardization, and multi-analyte capability. No single detection method meets all routine monitoring needs. HPLC-MS/MS offers the highest specificity and sensitivity for confirmation but is lab-bound, costly, and impractical for high-throughput screening. Rapid methods—including SERS, electrochemical biosensors, and lateral flow immunoassays—are field-deployable yet constrained by reproducibility, standardization, and multi-analyte capability. A practical strategy, therefore, combines rapid on-site screening with confirmatory chromatographic and MS analyses, an approach increasingly enhanced by machine learning [110].

In the context of SD detection, Brar et al. [111] developed a 2D-CNN explainable AI framework (based on DenseNet-169), achieving 99.99% accuracy for binary classification and 95.16% for multi-class classification of adulterated red chili powder. The model’s decision-making reliability was validated using Gradient-weighted Class Activation Mapping, providing interpretable visual evidence supporting the AI output [111]. Kuei and Chen [112] reported that an AI-assisted blockchain system offers a precise model for food quality monitoring and FF prevention, representing a key research path to enhance real-time decision-making capabilities under ambiguous conditions. Brar et al. [113] proposed an integrated DenseNet-169 CNN model with the ChoLevy-R optimizer to detect SD IV in red chili powder (95.78% accuracy), validated using FTIR spectroscopy coupled with SVM classification (96.55% accuracy) and PCA-based spectral peak identification. The Taiwan Food and Drug Administration (TFDA) has developed a deployable predictive model that integrates Random Forests, Naive Bayes, and deep learning algorithms to identify high-risk batches for SD residue testing [52]. Thereby enhancing prediction coverage and reducing unnecessary sampling inspections. Beyond monitoring and regulation, education programs have proven effective in increasing consumer awareness of FF risks. However, when applied to agricultural products, this approach uses tamper-proof methods to verify origin, journey, and manufacturing process (minimizing opportunities for adulteration) and to address issues such as revenue loss from recalls, food waste, and identification errors [89,104,108,110,112]. Thus, blockchain enhances transparency, mitigates fraud and contamination risks, strengthens consumer trust, and enables rapid recall response, establishing a new global standard for food system accountability [79,89,95,106]. Therefore, integrating lightweight CNNs, designed for IoT deployment with low power consumption, high efficiency, and real-time processing, with blockchain enhances food analysis precision, efficiency, and traceability, advancing testing technology innovation. [114,115].

Harmonizing global food safety standards enables multinational producers to achieve compliance more efficiently while fostering ongoing innovation in preservation techniques and safety interventions [107,108]. These case studies demonstrate that integrating environmental responsibility, data-driven strategies, and inclusive collaboration delivers tangible benefits across the food chain. At the same time, successful transformation hinges on co-creating value among producers, innovators, and consumers rather than on isolated action [89,104]. Weak penalty structures enable food manufacturers to treat fines as operational costs, prioritizing profits over compliance, making penalty reform a crucial component of SD prevention strategies [72]. However, spices remain indispensable to everyday cuisine. Yet the complexity and breadth of the modern food supply chain, coupled with multifaceted health risks and their intersection with diverse policy domains, mean that countries worldwide have developed unique food-related laws [46,72]. Conversely, robust food safety practices serve as competitive business strategies: failures can severely damage a company’s reputation, while compliance with international standards (e.g., participation in the Global Foodve) builds consumer trust and broadens Safety Initiati market access [38,116]. Influenced by customer demands and political pressures, exporters in developing countries often prioritize global food safety compliance over domestic regulatory challenges—as evidenced by Pakistan’s response to European RASFF notifications [116]. A principal oversight challenge in monitoring informal food markets, encompassing street food vendors, farmers’ markets, small-scale producers, and other operators lacking formal production premises, is the inherent difficulty of regulatory enforcement [38,73]. The lack of standardized international food safety regulations, combined with regulatory discrepancies and the difficulty of monitoring informal channels, significantly hinders effective oversight of SD adulteration [38,73].

International trade dynamics significantly influence SD adulteration patterns. A nation’s agri-food competitiveness depends on its engagement in international trade: trade-surplus nations gain stronger market positioning while deficit-dependent countries face vulnerabilities [105]. Agricultural trade diversifies dietary options, drives innovation through export competition, and stabilizes currency exchange rates [105]. Participants in the spice industry face rising compliance costs, driving the need for more advanced enforcement systems and the adoption of smart technology [89,110]. However, stricter food safety regulations, including zero-tolerance policies for SDs, raise trade costs by intensifying inspection requirements, causing shipment delays, and triggering border rejections [117]. Therefore, natural selection was once considered the sole form of evolution, much as current food safety regulations force companies to adapt to complex requirements, leading to a “survival of the fittest” scenario [90]. Tightening trade regulations raises costs for industry stakeholders and consumers, primarily through expanded testing requirements and border rejections of exports flagged for FF or adulteration [117]. Spice industry participants are especially affected. To ensure trade compliance, the industry must adopt smart technologies, strengthen fraud prevention, update FF and adulteration databases, and conduct annual risk analyses [106,117].

## 4. Potential Sources of Pollution

### 4.1. Spice Industry Overview and Market Structure

Spices and herbs have profoundly shaped human culture, trade, and exploration, demonstrated by their centuries-old use in religious rituals, traditional medicine, perfumery, and cosmetics. In modern food systems, they serve three evidence-supported functions: flavor enhancement, antimicrobial shelf-life extension, and delivery of bioactive compounds linked to potential health benefits, though causal clinical evidence remains limited for most claims [47,48,78]. The global spices and seasonings market remained at USD 29.9 billion in 2025, with an estimated CAGR of 4.53%, and is expected to reach USD 45.1 billion by 2034 [118]. Spices and herbs are used primarily in food manufacturing (75–80%), followed by retail (15–25%) and food service (5–10%) [119]. Demand is driven by consistent household consumption, the expansion of exotic foodservice offerings, and the widespread use of processed foods. At the same time, cleaner labels and simpler ingredient blends sustain long-term growth without significant fluctuations [118]. The therapeutic properties of spices and herbs have expanded the global market, enabling large-scale cultivation and trade of diverse plant parts—including roots, stems, bark, leaves, flowers, berries, fruits, and seeds [47,48,77,120].

The spice industry’s vulnerability to SD adulteration is structurally embedded in its supply chain design. Hoffman et al. [47] documented that spice-producing regions frequently experience political and economic instability compounded by lengthy distribution chains involving multiple intermediaries, conditions that facilitate contamination and adulteration. The journey of spices from cultivation to retail involves collectors, brokers, traders, agents, exporters, and processors, each playing essential roles in product delivery, yet also representing potential vulnerabilities to adulteration [47]. In particular, outdoor drying exposes the product to contamination from dust, sand, soil, or insects, while inadequate or absent refrigeration during transportation and storage further compromises quality [67]. Sadef et al. [67] also reported that some farmers or retailers might intentionally blend contaminated spices with uncontaminated ones to maximize their profits.

### 4.2. SD Contamination Pathways and Environmental Adsorption Properties

**Figure 3:**
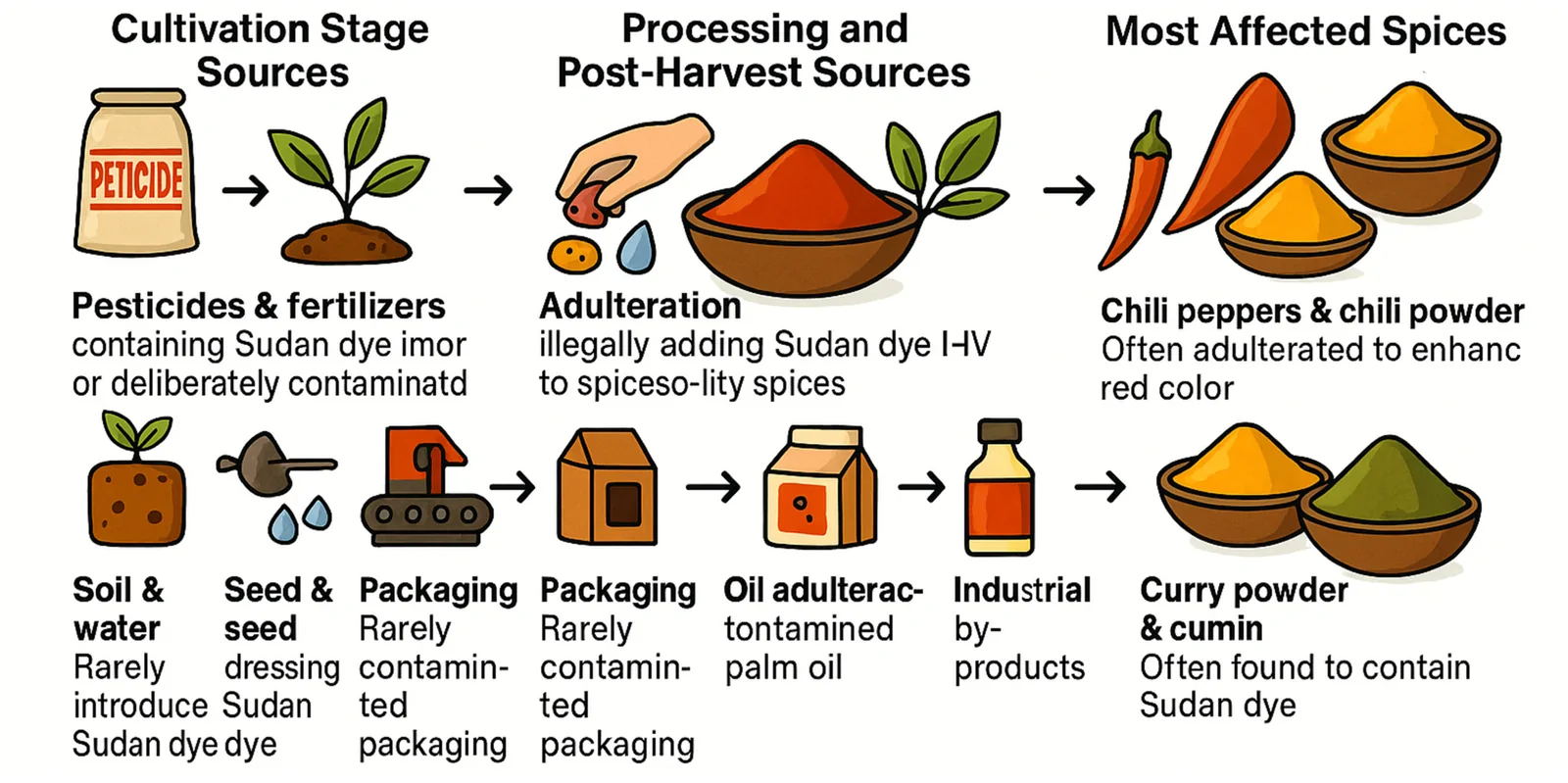
Contamination pathways for Sudan dye (SD) in spices: environmental migration and deliberate adulteration. It illustrates the contamination pathways and adulteration mechanisms by which SDs enter spice matrices. The diagram depicts two primary pathways: (1) environmental migration, where SDs from agricultural inputs (pesticides, contaminated fertilizers) or soil/water sources are absorbed by pepper plants and translocated through vascular tissues; and (2) deliberate adulteration, where industrial-grade SD is directly added to harvested spices (whole or ground chili, paprika, curry blends) to enhance color. The figure also highlights the role of processing steps (drying, grinding, blending) in distributing SD uniformly within spice products, making detection more challenging as the dye becomes homogeneously mixed. This dual-pathway model underscores that SD contamination may arise from both unintentional environmental exposure and intentional adulteration, necessitating different prevention strategies for each pathway.

Wu et al. [121] investigated the primary sources of SD I contamination in chili peppers, revealing that the compound originates principally from agronomic materials (pesticides and fertilizers) rather than direct soil contamination. The distribution of SD within the plant exhibits a progressive decrease from stems to leaves, then to fruits or roots (Figure 3).

**Figure 4:**
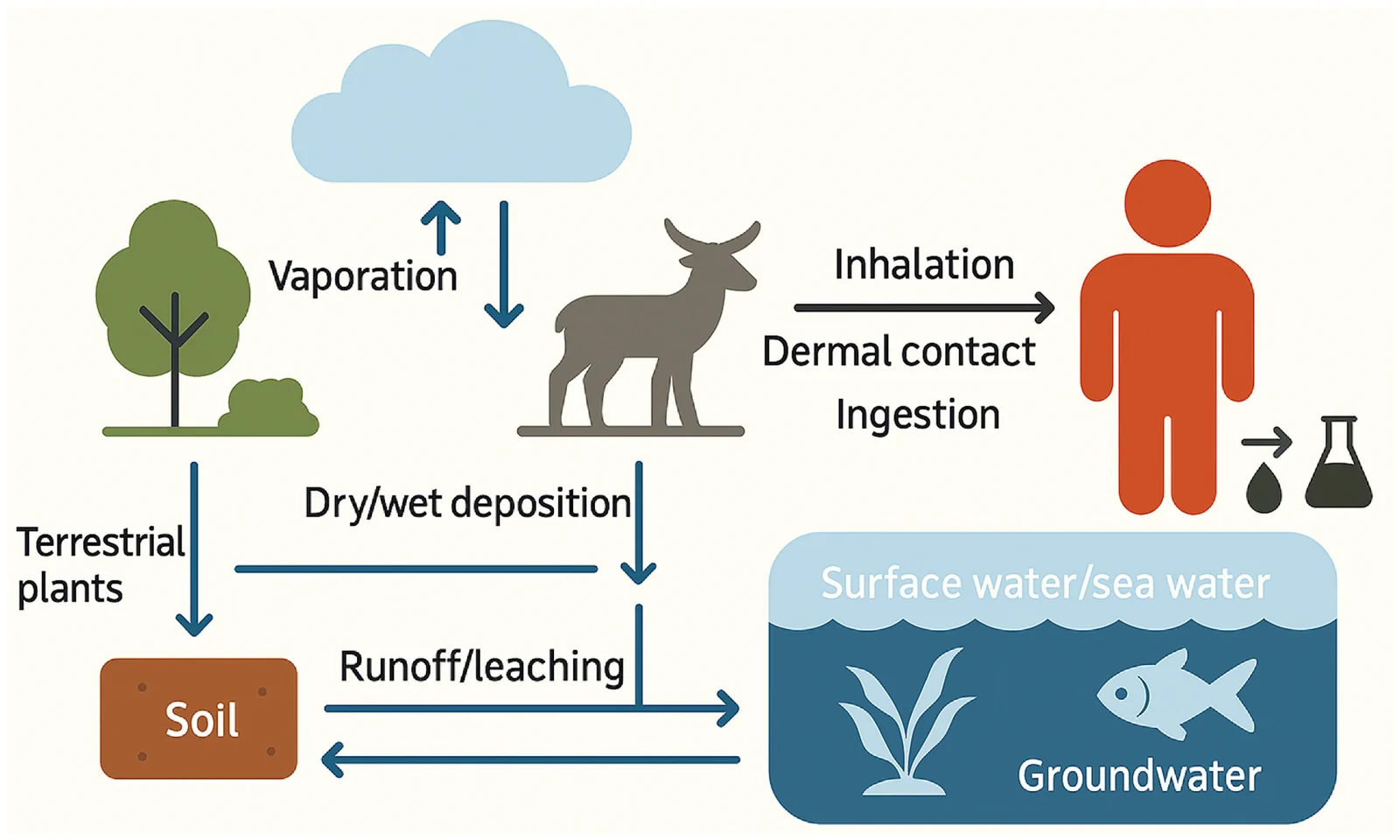
Adsorption characteristics of Sudan dyes (SDs) I–IV in a coastal soil system and their projected biogeochemical cycling. It shows adsorption behavior and projected biogeochemical cycling of SDs I–IV in coastal soils. SD II and IV, more lipophilic—sorb more strongly to soil particles, reducing mobility and increasing persistence. In contrast, SD I and III show adsorption plateaus at high concentrations, indicating saturation or competitive desorption that may enhance mobility under certain conditions. This differential behavior has implications for contamination risk assessment: SD II and IV are more likely to persist in soil adjacent to spice processing or drying facilities, while SD I and III may migrate more readily into groundwater.

The lipophilic nature of SDs enables rapid adsorption and accumulation in soil and facilitates their migration into water bodies, where they can disrupt biogeochemical cycles and enter aquatic ecosystems [122]. Specifically, the adsorption isotherms of SD I–IV follow the Langmuir model, in which adsorption capacity increases [122]. Notably, SD II and IV exhibit greater affinity for soil adsorption than SD I and III. In contrast, SD I, III, and IV exhibit abnormal adsorption fluctuations at high concentrations, highlighting the need to address their potential contamination and associated risks (Figure 4).

A one-month study showed that SD I–IV induced time-dependent effects on soil: initial enzyme suppression; mid-phase stimulation of key bacterial groups, followed by inhibition at high concentrations; and late-phase declines in microbial richness alongside increased biodiversity [123]. The common practice of using undeclared or substituted ingredients in spices, driven by economic motives, risks cross-contamination and necessitates strict safety protocols and clear labeling in the food industry to mitigate hazards [76]. Consequently, these factors provide fertile ground for corruption and other activities that may enable fraud, as described in Section 3. However, the current trend shows that slowing international demand has led to higher yields but lower prices for Indian capsicum and paprika crops [118]. Overlapping factors included: (1) reduced planting areas; (2) stricter international quality inspections due to contamination (FF) incidents; (3) the growing trend of functional blended spices meeting health demands; (4) improved processing techniques enhancing quality and profitability, and (5) food safety compliance (HACCP and origin traceability) becoming critical factors [118].

### 4.3. Prevention Measures and Supply Chain Management Strategies

To effectively mitigate fraud risks, food manufacturers must gain a comprehensive understanding of each stage of the spice supply chain, as its extended, intricate nature heightens vulnerability [47]. Apart from verification and testing, business operations are essential means: (1) streamline the supply chain or implement robust preventive controls at each node; (2) build proactive supplier partnerships, strengthen oversight, and gain direct insight into agricultural practices to identify vulnerabilities; (3) use forensic auditing to assess supplier finances and material flows; (4) apply mass balance analysis to detect input-output discrepancies—and economic intelligence to uncover large-scale origin misrepresentation (e.g., sanctions evasion via transshipment); and (5) cross-check certification documents against authoritative databases (e.g., USDA Organic Integrity Database) to verify organic spice claims [47,82]. The Rotterdam Convention’s “Prior Informed Consent” procedure provides a mechanism for countries to control the import of hazardous chemicals, including industrial dyes such as Sudan compounds, by requiring exporting nations to comply with importing countries’ decisions [103]. This framework has been empirically validated as effective in reducing trade volumes of regulated chemicals [103].

Moreover, large-scale producers adopt vertically integrated supply chain models: owning farms or contracting growers to cultivate materials while overseeing agronomic practices and harvesting processes [99]. This approach safeguards product quality and mitigates price volatility, allowing farmers to secure higher incomes while simultaneously increasing profits by eliminating intermediary costs [99]. Mitigating the risk of adulterated spices requires a proactive approach: building robust supplier partnerships, ensuring supply chain transparency, and advancing detection technologies, including LC-MS, X-ray fluorescence, DNA barcoding, clustered regularly interspaced short palindromic repeats, quantitative real-time Polymerase Chain Reaction (qPCR), and next-generation sequencing (NGS), as detailed in Section 1 [38,77,78,106,119,124]. Following the recommendations of Velazquez et al. [77], it is also necessary to implement on-site or convenient testing technologies for educated consumers who wish to verify the content of food labels. Optimizing a sustainable closed-loop supply chain network, integrating recycling and emissions reduction through a multi-objective mixed-integer linear programming framework, offers a quantitative route to reduce waste and pollutant emissions along the spice value chain [125]. The report identifies vendor lock-in as a key risk of cloud platforms, even as they ease deployment, thereby requiring industry-specific adaptation [125]. Automotive and electronics firms readily adopt them; the fast-moving consumer goods, food, and beverage sectors face greater challenges [125]. However, organizations need robust policies and institutional frameworks to harness transformative digital technologies (such as AI, IoT, blockchain, big data analytics, ML, and advanced robotics), while managing trade-offs [126]. These technologies enhance efficiency, transparency, and sustainability in socio-technical systems.

Consumers and manufacturers rely on nationally or regionally established ADIs for legal additives and for identifying hazardous toxins to ensure food safety. Under realistic dietary conditions, regular, sustained spice consumption—long-term exposure may involve unauthorized additives, deliberately added synthetic SDs, environmental contaminants, pesticide residues, and trace metals [48]. While it may not pose an immediate health risk, prolonged exposure of this nature can contribute to long-term health consequences. Another aspect to consider, despite advances in food supply chain management, is that industrial activities, agriculture, and improper handling can also introduce contamination into the food chain through heavy metals, pharmaceuticals, and pesticide residues [37]. Therefore, Ghuniem et al. [48] recommend broadening the current risk assessment framework to incorporate modeling of cumulative exposure and chronic intake, thereby providing a more accurate representation of actual dietary patterns and multi-pollutant interactions. It is worth noting that ensuring the authenticity of the global food supply is an essential moral and international task [106]. Hence, this is crucial for public trust, health equity, and environmental sustainability, and requires comprehensive systems aligned with the Global Food Safety Initiative standards and collective responsibility [106,127].

## 5. Conclusions

This review demonstrates that the persistence of SDs’ adulteration reflects fragmented detection, uneven regulation, and insufficient enforcement rather than a knowledge gap. An integrated strategy that combines cost-tiered screening methods with confirmatory chromatography, standardized regulatory protocols, and multi-stakeholder capacity building is more effective than isolated measures in suppressing FF. For practice, laboratories should adopt tiered frameworks that deploy low-cost rapid screening at production points and border crossings, escalating to definitive analysis only upon positive hits. At the same time, industry operators can reduce vulnerability through supplier verification programs and targeted employee training on FF awareness. Blockchain- and IoT-enabled traceability improves supply chain transparency, but it falls short unless data quality, system interoperability, and implementation cost are addressed, especially for resource-constrained actors. Policy actions should prioritize risk-based enforcement in vulnerable supply segments, harmonized regulatory standards across jurisdictions to close arbitrage loopholes, and public–private partnerships to share traceability infrastructure costs among governments, industry, and international organizations. Future research should (1) validate AI-enhanced screening platforms through large-scale field trials assessing real-world detection rates and false-positive frequencies; (2) conduct comparative analyses of blockchain traceability systems to establish data standardization and interoperability protocols; (3) from a theoretical standpoint, integrating the crime triangle with demand-side analysis provides a more comprehensive lens for understanding FF than any single criminological framework applied in isolation; (4) perform longitudinal evaluations of harmonized regulatory frameworks across jurisdictions; and (5) investigate demand-side interventions, including consumer education campaigns and labeling regulations, to reduce market incentives for appearance-enhancing FF. Overall, this review proposed prioritized recommendations: for researchers, multimodal, transferable analytical platforms; for regulators, harmonized zero-tolerance enforcement and transboundary cooperation; for industry, HACCP- or FSA-based vulnerability assessments and traceability; for consumers, targeted education and demand for certified or traceable products.

## Data Availability

No new data were created or analyzed in this study. Data sharing is not applicable to this article.

## Abbreviations

The following abbreviations are used in this manuscript:

SD	Sudan dyes
FF	Food fraud
AZD	Azo dyes
ADI	Acceptable Daily Intake
CAGR	Compound annual growth rate
ADHD	Attention deficit hyperactivity disorder
IARC	International Agency for Research on Cancer
EFSA	European Food Safety Authority
NTD	New Taiwan dollars
LC-MS/MS	Liquid chromatography-tandem mass spectrometry
ML	Machine learning
CNN	Convolutional neural network
RASFF	Rapid Alert System for Food and Feed
EMA	Economic Motivated Adulteration
RFID	Radio frequency identification
AI	Artificial intelligence
IoT	Internet of Things
SDGs	Sustainable Development Goals
BFTS	Blockchain-based food traceability systems
GPFH	General Principles of Food Hygiene
Codex	Codex Alimentarius
HACCP	Hazard Analysis Critical Control Point
FTIR	Fourier transform infrared
PCA	Principal component analysis
TFDA	Taiwan Food and Drug Administration
qPCR	Quantitative real-time Polymerase Chain Reaction
NGS	Next-generation sequencing
